# Extracellular microvesicles and invadopodia mediate non-overlapping modes of tumor cell invasion

**DOI:** 10.1038/srep14748

**Published:** 2015-10-13

**Authors:** Alanna E. Sedgwick, James W. Clancy, M. Olivia Balmert, Crislyn D’Souza-Schorey

**Affiliations:** 1 Department of Biological Sciences, University of Notre Dame, Notre Dame, IN 46556-0369, USA

## Abstract

Tumor cell invasion requires the molecular and physical adaptation of both the cell and its microenvironment. Here we show that tumor cells are able to switch between the use of microvesicles and invadopodia to facilitate invasion through the extracellular matrix. Invadopodia formation accompanies the mesenchymal mode of migration on firm matrices and is facilitated by Rac1 activation. On the other hand, during invasion through compliant and deformable environments, tumor cells adopt an amoeboid phenotype and release microvesicles. Notably, firm matrices do not support microvesicle release, whereas compliant matrices are not conducive to invadopodia biogenesis. Furthermore, Rac1 activation is required for invadopodia function, while its inactivation promotes RhoA activation and actomyosin contractility required for microvesicle shedding. Suppression of RhoA signaling blocks microvesicle formation but enhances the formation of invadopodia. Finally, we describe Rho-mediated pathways involved in microvesicle biogenesis through the regulation of myosin light chain phosphatase. Our findings suggest that the ability of tumor cells to switch between the aforementioned qualitatively distinct modes of invasion may allow for dissemination across different microenvironments.

The ability of cells to invade into and traverse the extracellular environment is a prerequisite for tumor cell dissemination and metastasis^1^,^2^. The deregulation of cell-cell and cell-matrix interactions, together with matrix proteolysis to enable cell movement through the extracellular matrix^3^,^4^,^5^, underlies some of the most unfavorable events early in cancer progression. A significant body of work has demonstrated that individual tumor cells can adopt and readily switch between two different inter-convertible phenotypes during movement; a mesenchymal phenotype with flat and elongated morphology and an amoeboid phenotype with a more rounded and high blebbing morphology^6^,^7^,^8^. Consequently, the mechanisms utilized by individual tumor cells with either phenotype to invade its surrounding tissues, and the cell’s ability to switch between these phenotypes, are likely to critically influence tumor dissemination during invasion and metastasis.

Invadopodia are protease-rich membrane protrusions formed at the adherent surface of invading tumor cells. These protrusions have been documented as foci for localized matrix proteolysis and their role in facilitating cell invasion is well-characterized^9^. A variety of components are recruited to sites of invadopodia formation, including proteins necessary for actin and membrane remodeling as well as matrix proteolysis. Invadopodia formation requires the activation of Rac1 and subsequent downstream signaling^10^,^11^,^12^,^13^,^14^,^15^,^16^.

In recent years, another cell structure has garnered increased interest for its potential to degrade matrix, namely, extracellular tumor cell-derived microvesicles (TMVs). Formed from membrane blebs at the plasma membrane, TMVs are selectively enriched with molecular cargo including proteases, and are pinched from the membrane via acto-myosin-based contraction that is at least in part mediated by the small GTP binding protein ARF6^17^. Once discounted as merely cell debris, it is now understood that these shed membrane vesicles can condition the tumor microenvironment in varied ways, including matrix proteolysis to facilitate cell invasion^18^,^19^,^20^.

TMVs are distinct from exosomes, another extracellular vesicle released from tumor cells and other cell types^17^,^21^. Exosomes range from 50–80 nm in diameter whereas TMVs are more heterogeneous in size and larger, ranging from a few hundred nanometers to a few microns in diameter. TMVs form by the outward budding of the plasma membrane, whereas exosomes are released by fusion of the limiting membrane of multivesicular bodies with the cell surface^22^. TMVs share many characteristics with oncosomes, first described as the extracellularly shed non-apoptotic blebs induced by the deletion of the actin nucleating protein, DRF3/Dia2^23^.

Here we show that TMVs and invadopodia facilitate qualitatively distinct modes of cell invasion. Invadopodia formation and high levels of Rac1 activity accompany mesenchymal movement on firm matrices, whereas amoeboid motility, which predominates on more deformable and complaint matrices, requires Rho-regulated actomyosin-based contraction and is accompanied by TMV shedding. In addition, we demonstrate that competitive signaling through RhoA and Rac1 are integral for the formation of these distinct invasive structures and allow for phenotypic plasticity during invasion. We also unravel additional Rho-mediated pathways that, in parallel with ARF6, support microvesicle biogenesis through the regulation of myosin light chain activity. These studies potentially impact the design of therapeutic agents aimed at attenuating tumor invasion.

## Results

### Extracellular matrix compliance guides the choice of invasive structures

**Figure 1:**
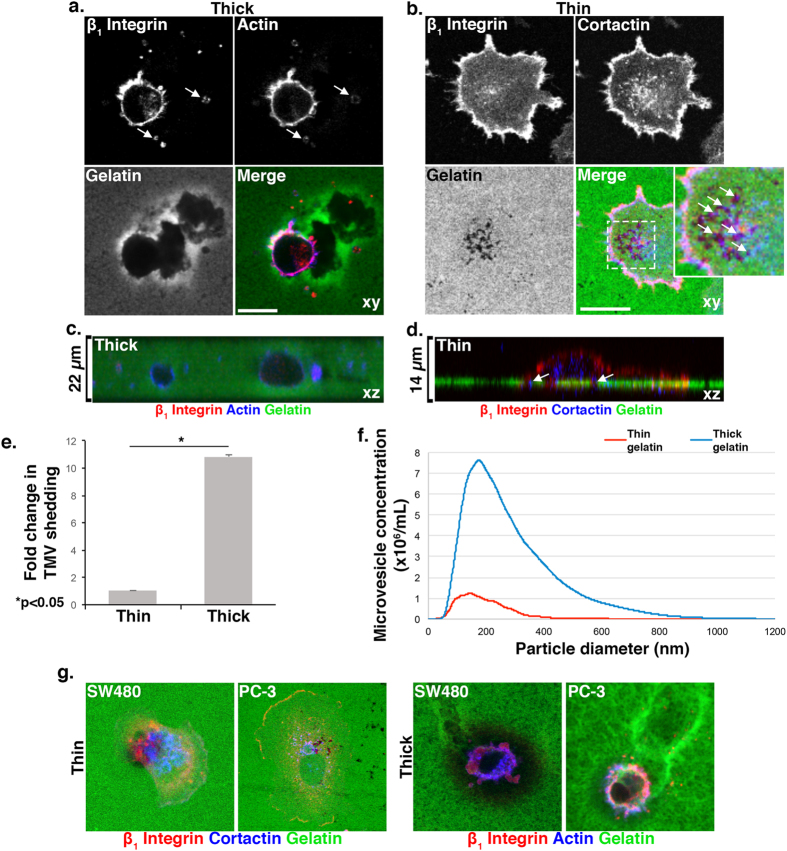
Matrix characteristics modulate microvesicle release. (**A**) LOX cells were grown on thick FITC-gelatin matrix prior to fixation and staining to visualize β_1_ integrin (red) and actin (blue). Arrows indicate microvesicles. XY axes shown. (**B**) LOX cells were grown on thin FITC-gelatin matrix prior to fixation and staining to visualize β_1_ integrin (red) and cortactin (blue). Enlarged panel of merged image shown with arrows to indicate colocalization of cortactin at sites of degradation. XY axes shown. (**C**) LOX cells were grown on thick FITC-gelatin matrix and stained as in A. XZ axis shown. (**D**) LOX cells were grown on thin FITC-gelatin matrix and stained as in B. Arrows indicate colocalization of cortactin at sites of degradation. XZ axis shown (**E**) Microvesicles released from cells grown on thin or thick matrices were quantified by microscopy, as described in Methods. 140 cells were counted per condition and the data shown represents the fold change in average number of TMVs shed per cell. Error bars represent the standard error of the mean. (**F**) Conditioned media was harvested from 50,000 cells grown in thin or thick FITC-gelatin and analyzed by nanoparticle tracking as in the Methods. (**G**) SW480 and PC-3 cells were grown on thin or thick FITC-gelatin and demonstrate invadopodia formation or microvesicle release, respectively. Cells on thin gelatin were stained for β_1_ integrin (red) and cortactin (blue), and those on thick gelatin were stained for β_1_ integrin (red) and actin (blue).

To better elucidate the roles of microvesicles and invadopodia during cell invasion, the invasive melanoma cell line LOX, adept at forming both invasive structures^17^,^24^, was plated onto fluorescently-labeled, denatured collagen (gelatin). As has been previously reported, cells plated on >20 μm thick, deformable matrix, adopted a rounded, blebbing, amoeboid morphology^5^,^7^,^25^. When placed atop the matrix, the cells embedded themselves within it as they invaded. Labeling of these cells for proteins at the cell surface, such as β_1_ integrin, showed that these rounded amoeboid cells shed abundant TMVs, many of which are contained within the surrounding matrix. (Fig. 1a). Due to its consistent localization at the surface of TMVs, β_1_ integrin was used to mark these vesicles in all subsequent immunofluorescence studies. Notably, membrane protrusions characteristic of invadopodia were not detected at the adherent surfaces of these cells. Comparable TMV release was noted in cells invading a thick, deformable, fibrillar collagen I matrix as observed by confocal reflection microscopy (Supplementary Fig. S1).

When LOX cells were plated onto thinly coated (≤5 μm) coverslips, resulting in a more firm or rigid substrate as the gelatin is closely apposed to an inflexible surface, the majority of cells adopted a flattened, spread, mesenchymal morphology as previously reported^7^,^26^ (Fig. 1b). These cells formed degradation patterns typical of invadopodia-mediated degradation, namely, pinpoint puncta of matrix degradation directly beneath cells co-localizing with ventral protrusions containing the invadopodia marker cortactin^9^. Fig. 1c,d show released microvesicles and invadopdia, respectively, along the z axis. As evident from these images, only a limited number of cells on thin matrices form membrane blebs and the number of microvesicles released is very minor in comparison to cells in thick, deformable matrix. This was confirmed both by counting the number of TMVs embedded within the matrix (Fig. 1e) as well as by nanoparticle tracking analysis of vesicles isolated from the media of cells invading thick gelatin substrates (Fig. 1f). Isolated microvesicles used for biochemical and nanoparticle tracking analyses were also fixed onto coverslips and stained for β_1_ integrin (Supplementary Fig. S2). In addition to melanoma cells, these observations of invasive cell morphologies were consistent across other invasive tumor cell lines, including colon and prostate tumor cell lines (Fig. 1g).

**Figure 2:**
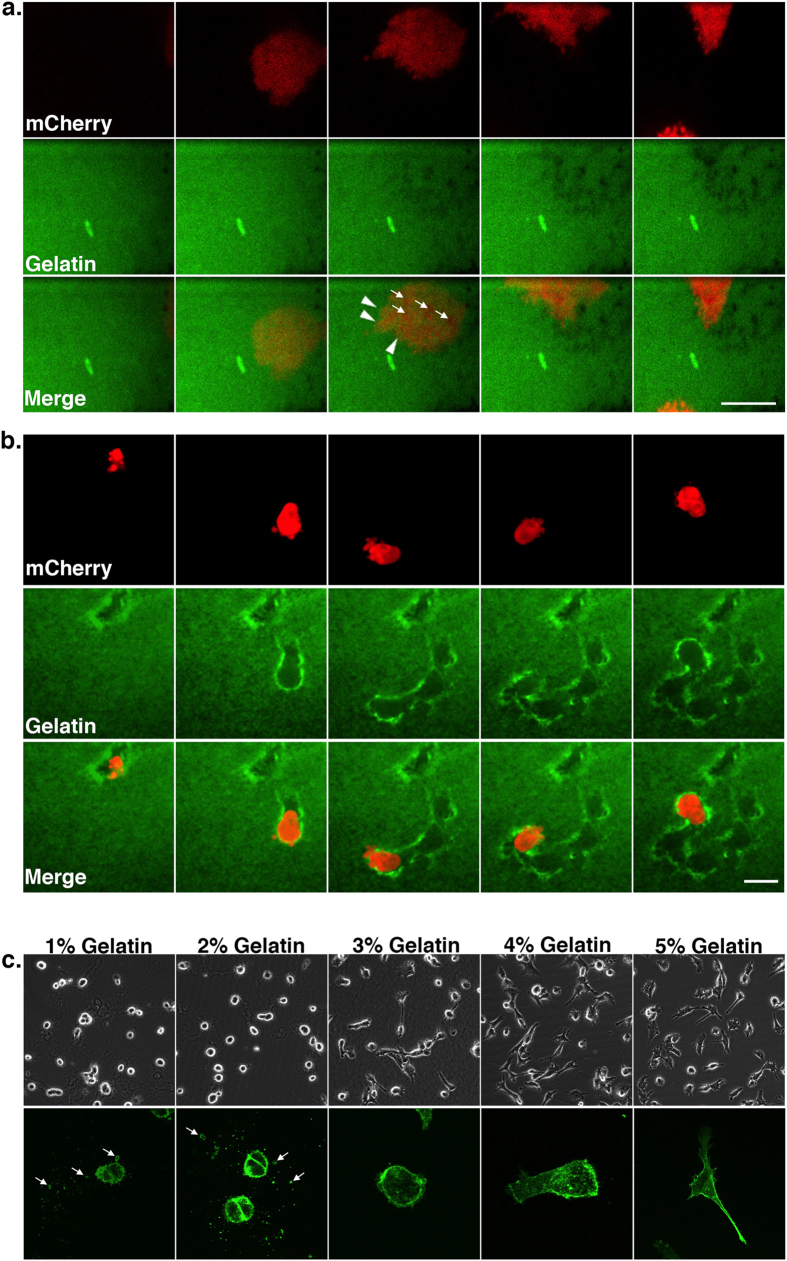
Matrix stiffness modulates cell morphology and mode of invasion. (**A**) Cells transiently transfected to express cytoplasmic mCherry were grown on thin FITC-gelatin and imaged live using confocal fluorescence microscopy. A sequence of time-lapse images taken from Supplementary movie 1 is shown. Arrowheads indicate the leading edge of the cell, and arrows indicate forming proteolytic puncta. Scale bar = 20 μm. (**B**) Cells transiently transfected to express cytoplasmic mCherry were grown in thick FITC-gelatin and imaged as in A. Scale bar = 20 μm. (**C**) Cells were grown on approximately 50 μm of gelatin in concentrations from 1 to 5%. The cells are shown both live using phase contrast microscopy (top), and fixed at higher magnification after staining for β_1_ integrin (green, bottom). Arrows indicate shed microvesicles.

Live cell imaging revealed distinct invasion patterns depending upon matrix conditions. On thin matrices, cells transiently expressing the cytoplasmic red fluorescent protein mCherry were flattened and spread, “crawling” across the surface and leaving small puncta of degraded matrix from invadopodia-dependent proteolysis in their wake (Fig. 2a, Supplementary movie 1). However, when plated on more compliant beds of >20 μm thick ECM, the cells were observed to “drill” blebbing surface protrusions into the matrix at the invasive leading front and subsequently tunnel through the matrix creating degradation trails tens of micrometers in length (Fig. 2b, Supplementary movie 2).

In order to discern phenotypic effects mediated by matrix compliance versus matrix thickness, we plated cells onto glass coverslips that were coated with matrices approximately 50 μm thick, but with increasing concentrations of gelatin in order to modulate substrate stiffness^27^. We found that on matrices containing concentrations of 1% to 2% gelatin, cells readily adopted a round morphology and released TMVs into the ECM (Fig. 2c, Supplementary Fig. S3). As gelatin concentration and matrix stiffness are further increased the cells became flattened and elongate, and lose the ability to embed in the matrix and shed microvesicles. A sharp increase in matrix stiffness has been characterized as gelatin levels increase from 2.5% to 5%^27^, which likely explains this alteration in cell behavior. At the higher concentration of matrix, this flat cellular phenotype mimics that exhibited by cells plated onto a ≤5 μm layer of 2% gelatin. As stated earlier, the “thin” layer of 2% matrix, which is closely apposed with an inflexible glass coverslip, presents these cells with a rigid extracellular environment. On the other hand, 2% gelatin matrix greater than 20 μm in depth, henceforth referred to as “thick”, encourages the adoption of an amoeboid morphology. As such, subsequent experiments were performed by varying the thickness of 2% gelatin to alter cell behavior as this offers some benefits, including enhanced immunofluorescence staining and image acquisition, both of which are more challenging at high matrix concentrations.

### Rac1 activity antagonizes microvesicle shedding

**Figure 3:**
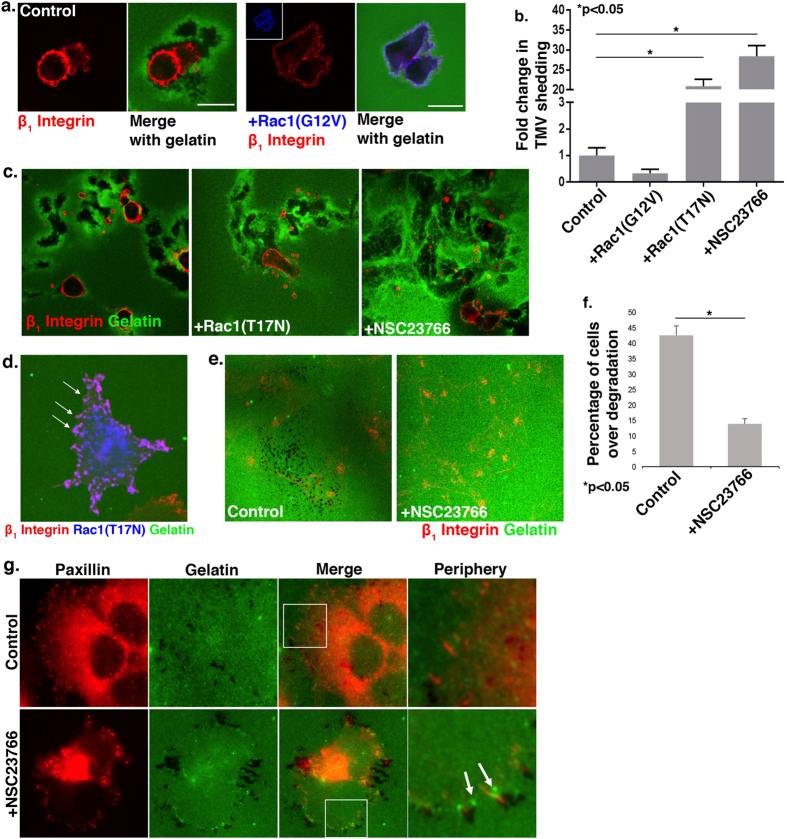
Rac1 activation suppresses TMV shedding. (**A**) LOX cells transiently transfected with T7-Rac1(G12V) were seeded on a thick layer of FITC-gelatin prior to fixation and staining to visualize β_1_ integrin (red) and the T7 tag (blue). Scale bars = 20 μm. (**B**) Microvesicles released from the cells under experimental conditions described in A and C were quantified by microscopy, as described in the Methods. 140 cells were counted per condition and the data shown represents the fold change in the average number of TMVs shed per cell. Error bars represent the standard error of the mean. (**C**) Cells were transiently transfected with T7-Rac1(T17N) or treated with 50 μM NSC23766 and grown on a thick layer of FITC-gelatin prior to fixation and staining to visualize β_1_ integrin (red). (**D**) Cells transiently transfected with T7-Rac1(T17N) were grown on thin FITC-gelatin prior to fixation and staining to visualize the T7 tag (blue) and β_1_ integrin (red). An optical section along the dorsal cell surface is shown and transfected cells displayed small plasma membrane blebs (arrows), however TMV release was not observed. (**E**,**F**) Cells were treated with 50 μM NSC23766 and grown on a thin layer of FITC-gelatin prior to fixation and staining to visualize β_1_ integrin (red). Optical sections along the adherent surface of the cells are shown. 100 cells were counted per condition and the percentage of cells over degraded matrix was quantified. The error bars represent the standard error of the mean. (**G**) Cells were treated with 50 μM NSC23766 and grown on thin FITC-gelatin prior to fixation and staining to visualize paxillin (red) as a marker of focal adhesions. Magnified areas along the cell periphery are shown. Unlike untreated control cells (upper panel), cells treated with Rac1 inhibitor (lower panel) display increased focal contacts that appear to pull on the matrix, which gets doubled-over resulting in more intense FITC puncta (arrows). This is distinct from invadopodia-mediated proteolysis in the upper panel.

Since the formation of invadopodia and cell invasive capacity are associated with elevated levels of Rac1 activation^10^,^11^,^12^,^13^,^14^,^15^,^16^, we questioned whether Rac1 activity also promoted the formation of invasive microvesicles. We first examined the effect of sustained Rac1 activation via expression of the Rac1-GTP mutant, Rac1(G12V), on microvesicle release. On a thick, compliant matrix, Rac1(G12V)-expressing cells are more flattened and elongated, often displaying thin filopodial protrusions rather than adopting rounded and amoeboid morphologies as in the control cells (Fig. 3a). Strikingly, their ability to form TMVs is abrogated (Fig. 3a,b). Moreover, these cells display no detectable invadopodia, which is in marked contrast to the increase in invadopodia-mediated invasion which has been shown to occur when Rac1 is activated in cells on firm matrices^16^. Thus, while antagonizing microvesicle shedding, Rac1 activation supports invadopodia formation but only on firm matrices.

We assessed the effects of inhibiting Rac1 activity by treating cells with the small molecule inhibitor NSC23766, which selectively prevents the activation of Rac1^28^, or transient transfection of cells with the dominant-negative Rac1 mutant, Rac1(T17N). When plated onto a thick gelatin matrix, cells treated with NSC23766 or those expressing Rac1(T17N) showed a dramatic increase in microvesicle shedding compared to control untransfected cells or those transfected with the vector alone (Fig. 3b,c). Notably, when cells expressing Rac1(T17N) were grown on a thin and firm matrix, they retained flat morphologies, often decorated with large numbers of small membrane blebs which remain attached to the cell surface (Fig. 3d). These observations support the contention that downregulation of Rac1 activity promotes TMV release but only on compliant surfaces, as rigid matrices are not conducive to TMV release. Further, on thin and rigid matrix, Rac1 inhibited cells also exhibited little to no invadopodia-mediated matrix proteolysis (Fig. 3e,f), corroborating the published literature^10^,^11^,^12^,^13^,^14^,^15^,^16^,^29^. Although degradation of the matrix was sometimes observed and mostly around flat, spread cells, closer examination revealed patterns that did not correlate with invadopodia. Inhibitor treated cells demonstrated the clustering of paxillin into the formation of robust, elongated focal contacts at the cell periphery (Fig. 3g). The areas of degradation are often bordered by an area of bright matrix, likely because it is doubled-over, and filaments of the matrix are observed to be pulled back to the edge of the cell. Whereas untreated cells display diffuse, small paxillin puncta across the ventral surface, inhibitor treated cells display highly clustered, large, mature focal contacts at the periphery that pull on the matrix. It is unclear, and perhaps unlikely, that focal adhesion-mediated proteolysis, as has been characterized to occur in certain cell types^30^, is responsible of the matrix rupture observed here. This data supports existing literature that unlike invadopodia, Rac1 is not required for the formation of focal adhesions, which has been characterized as a Rho-driven process^31^.

### Rac1 and RhoA are differentially activated in cells invading firm versus compliant matrices

**Figure 4:**
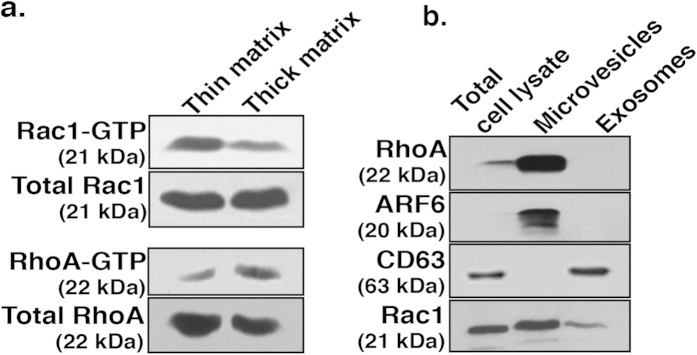
Matrix properties guide the activation of Rac1 and RhoA. (**A**) Cells were grown on a thin or thick gelatin matrix prior to incubation with PAK-PBD or Rhotekin-RBD beads in effector pulldown assays to assess the levels of active Rac1 and RhoA, respectively. A sample of the total cell lysate was blotted for total Rac1 or RhoA. (**B**) Microvesicles and exosomes were isolated from cells via serial centrifugation and lysed for western blotting. Equal amounts of protein were loaded for each fraction. ARF6 is shown as a marker for microvesicles and CD63 for exosomes. RhoA is enriched in shed microvesicles but is not present on exosomes. All western blot film images were cropped to show the proteins of interest, and all blots were run using the same experimental conditions.

In light of the above findings, we examined endogenous Rac1-GTP and RhoA-GTP pools on firm and compliant matrices. Biochemical examination using effector pulldown assays revealed that on thin, firm substrates, cells displayed elevated Rac1-GTP levels, whereas RhoA-GTP were elevated in cells collected from thick and compliant matrix (Fig. 4a). Further, microvesicles are highly enriched in RhoA when compared with the total cell lysate (Fig. 4b). While Rac1 was also found on isolated TMVs it was not enriched above the levels found in whole cell lysates. ARF6 was used to confirm the identity of the microvesicle fraction, and as previously reported^17^, ARF6 content is enriched in TMVs. While the inclusion of Rac1 in microvesicles is interesting, the presence of certain cargoes within TMVs yields limited information as to their roles in biogenesis, and at this time it is unclear what functions RhoA and Rac1 may play as microvesicle cargoes. Furthermore, we subsequently examined the exosome fraction released by these cells, using the tetraspanin CD63 to confirm exosomal identity^29^, and found that these structures are devoid of RhoA, but do contain relatively lower levels of Rac1 (Fig. 4b).

### RhoA/ROCK signaling promotes TMV biogenesis and suppresses invadopodia formation

**Figure 5:**
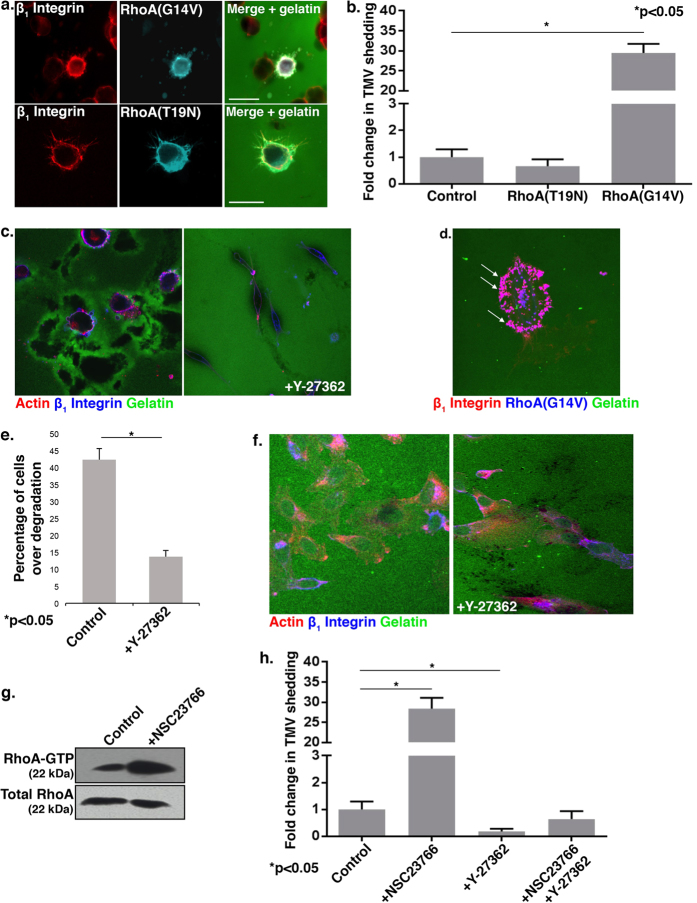
RhoA activation promotes TMV formation. (**A**,**B**) Cells transiently transfected with T7-tagged RhoA(G14V) or RhoA(T19N) were grown on a thick layer of FITC-gelatin prior to fixation and staining to visualize β_1_ integrin (red) and the T7 tag (pseudocolored cyan). Scale bars =20 μm. (**B**) Microvesicles released from cells were quantified as detailed in the Methods, and the data shown represents the fold change in the average number of TMVs shed per cell. Error bars represent the standard error of the mean. (**C**) Cells were grown on a thick layer of FITC-gelatin and treated with 10 μM Y-27632 prior to fixation and staining to visualize F-actin (red) and β_1_ integrin (blue). (**D**) Cells transiently transfected with T7-tagged RhoA(G14V) were grown on a thin layer of FITC-gelatin prior to fixation and staining for β_1_ integrin (red) and the T7 tag (blue). Cells expressing active RhoA form blebs (arrows) but do not release TMVs on firm matrix. (**E**,**F**) Cells were grown on a thin layer of FITC-gelatin and treated with 10 μM Y-27632 prior to fixation and staining to visualize β_1_ integrin (blue) and F-actin (red). 100 cells per experimental condition were counted, and the percentage of cells over proteolysed matrix was quantified. The percentage of invading cells over degradation per condition is shown. The error bars represent the standard error of the mean. (**G**) Cells were grown on a thick gelatin matrix in the presence of 50 μM NSC23766 prior to lysis and Rhotekin-RBD effector pulldown assay to assess the levels of RhoA-GTP. Total cell lysate was blotted for total RhoA. (**H**) Microvesicles released from cells grown in thick FITC-gelatin and treated with 50 μM NSC23766 (NSC) and/or 10 μM Y-27632 (Y) were quantified as detailed in the Methods. 140 cells were counted per condition and the data shown represents the fold change in the average number of TMVs shed per cell. Error bars represent the standard error of the mean. All western blot film images were cropped to show the proteins of interest, and all blots were run using the same experimental conditions.

Since RhoA is present on TMVs and amoeboid movement is associated with high levels of actin-based contractility^32^, we investigated the requirements for RhoA activation on TMV release. Expression of the constitutively-active mutant of RhoA, RhoA(G14V), dramatically increased microvesicle shedding in thick matrices (Fig. 5a,b). In stark contrast, when the dominant-negative RhoA(T19N) mutant was expressed, TMV formation was strongly diminished (Fig. 5a,b), showing an approximately 50% reduction. The majority of cells form thin filopodial protrusions, reminiscent of what is seen with the expression of constitutively-active Rac1. Likewise, cells treated with the Rho-associated protein kinase (ROCK) I/II inhibitor Y-27632 lose their ability to adopt amoeboid morphologies, instead maintaining a flatter and often elongated morphology while unable to form microvesicles (Fig. 5c). Interestingly, when cells expressing RhoA(G14V) were grown on a thin matrix, the cells exhibited a flattened morphology while forming extensive small plasma membrane blebs (Fig. 5d), which, notably, is very similar to the behavior cells with Rac1 activation inhibited. These are not, however, shed from the cell as microvesicles. In contrast, the inhibition of ROCK enhanced invadopodia formation on firm matrix (Fig. 5e), and the matrix was decorated with small, punctuate proteolyzed spots (Fig. 5f). Furthermore, NSC23766 treatment markedly increased the pool of endogenous RhoA-GTP in cells invading a thick matrix (Fig. 5g), indicating that down-regulating Rac1 activation also amplifies RhoA activation and supporting the hypothesis that Rac1 inhibition fosters TMV formation by promoting the activation of RhoA. To confirm this relationship, we found that ROCK inhibition blocked TMV release induced by treatment with the Rac1 inhibitor, NSC23766 (Fig. 5h).

Collectively, these studies show that tumor cells are able to switch between the use of microvesicles and invadopodia to facilitate invasion through extracellular matrix, and that this behavior is governed by antagonistic signaling through RhoA and Rac1, as well as the physical properties of the extracellular matrix.

### RhoA/ROCK signaling facilitates microvesicle shedding downstream of ARF6 activation and Rac1 downregulation

**Figure 6:**
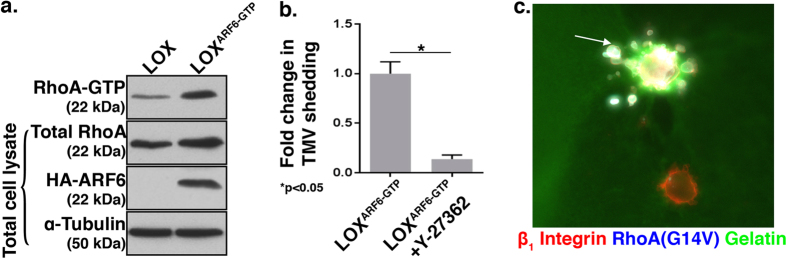
RhoA is activated downstream of ARF6 during TMV shedding. (**A**) LOX and LOX^ARF6-GTP^ were assessed for levels of RhoA-GTP using a Rhotekin-RBD effector pulldown assay. Cell lysates were also probed for total levels of RhoA and α-tubulin, and HA to confirm the identity of the HA-tagged ARF6 mutant. (**B**) Microvesicles released from LOX^ARF6-GTP^ and LOX^ARF6-GTP^ treated with the ROCK inhibitor Y-27632 were quantified by microscopy as detailed in the Methods, and the data shown represents the fold change in the average number of TMVs shed per cell. The error bars represent the standard error of the mean. **C.** LOX cells that stably express LOX^ARF6-GDP^ were transiently transfected to express T7-tagged RhoA(G14V) (arrow) and grown on a thick FITC-gelatin matrix prior to fixation and staining to visualize the T7 tag on RhoA (pseudocolored cyan) and β_1_ integrin (red). All western blot film images were cropped to show the proteins of interest, and all blots were run using the same experimental conditions.

As previously described, the activation of the small GTP-binding protein ARF6 enhances microvesicle release^17^. In cells wherein microvesicle formation is dramatically increased by the expression of constitutively-active ARF6, ARF6(Q67L), an increase in RhoA activation is noted (Fig. 6a). The inhibition of Rho kinase, which lies downstream of RhoA, abrogates ability of ARF6-GTP mutant expressing cells to adopt an amoeboid morphology or form microvesicles (Fig. 6b). These data indicate that Rho signaling occurs downstream of ARF6 to facilitate TMV shedding. In addition, RhoA(G14V) expression is able to rescue TMV shedding in cells expressing the dominant negative ARF6 mutant, ARF6(T27N) (Fig. 6c). Collectively, we have shown that ARF6 activation as well as Rac1 inhibition, can feed into pathways that prompt RhoA activation to facilitate TMV shedding.

### ROCK-mediated inhibitory phosphorylation of myosin light chain phosphatase promotes MLC activation

**Figure 7:**
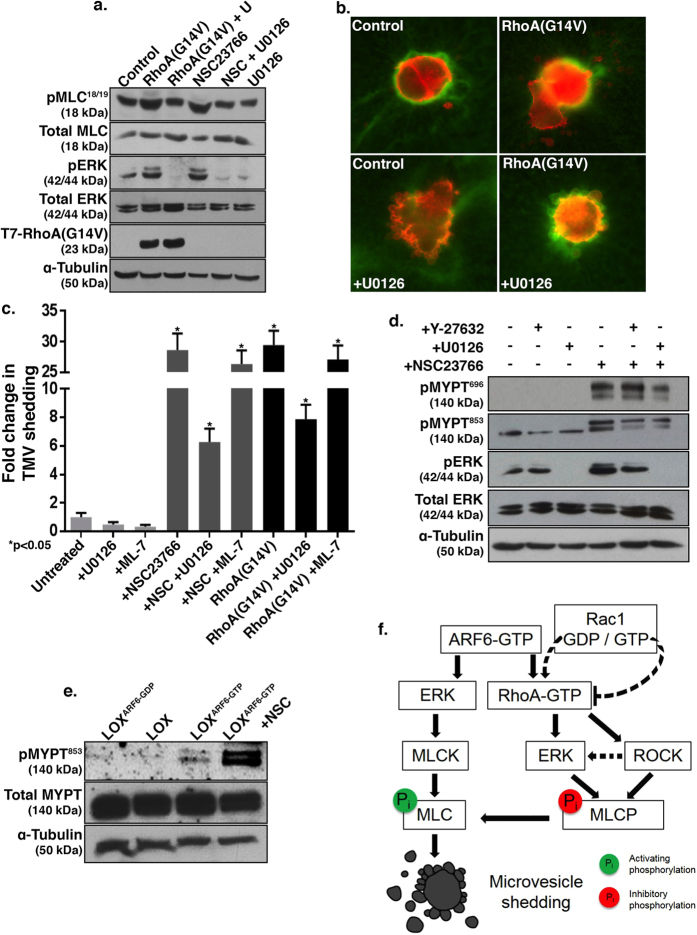
ERK and ROCK-mediated regulation of myosin light chain phosphatase during TMV shedding. (**A**) Untransfected cells or those transfected with T7-tagged RhoA(G14V) were grown on a thick gelatin matrix and treated with either 50 μM NSC23766 (NSC), 20 μM U0126 (U), or both, prior to western blotting for total and phospho-MLC^18/19^, total and phospho-ERK^202/204^, the T7 tag, and α-tubulin. (**B**) Untransfected cells and those transiently transfected with RhoA(G14V) were grown on a thick FITC-gelatin matrix and treated with 20 μM U0126 prior to fixation and staining to visualize β_1_ integrin (red). (**C**) Control cells and those transiently transfected to express RhoA(G14V) were grown on a thick FITC-gelatin matrix and treated with U0126, ML-7, or NSC23766, as indicated prior to fixation and staining to visualize β_1_ integrin and the T7-tagged mutants. Released microvesicles were quantified as described in the Methods, and the error bars represent the standard error of the mean. (**D**) Cells were grown on thick gelatin matrix and treated with U0126, Y-27632, NSC23766, or a combination thereof as indicated, prior to western blotting to assess phospho-MYPT1 Thr^696^, phospho-MYPT1 Thr^853^, phospho-ERK^202/204^, total ERK, and α-tubulin. (**E**) LOX, LOX^ARF6-GDP^, LOX^ARF6-GTP^, and LOX^ARF6-GTP^ treated with 50 μM NSC23766 (NSC) were grown on a thick gelatin matrix prior to lysis and western blotting for total and phospho-MYPT1^853^ and α-tubulin. (**F**) Schematic representation of signaling pathways that lead to MLC phosphorylation to promote TMV shedding. In addition to the ARF6-ERK-MLCK pathway previously described^17^, RhoA signaling also promotes TMV shedding by activation of ERK and ROCK leading to the inhibitory phosphorylation of the MYPT1 subunit of myosin phosphatase. ARF6 activation and Rac1 inhibition feed into pathways that promote RhoA activation. All western blot film images were cropped to show the proteins of interest. All protein gels were run using 12% acrylamide, except for MYPT1 blots which were run using 8% acrylamide.

Previous studies have demonstrated that MLC phosphorylation is required for the actomyosin contractility at the “necks” of TMVs and their subsequent abscission and shedding from the plasma membrane^17^. MLCK (MLC kinase) mediates this increase in MLC phosphorylation, downstream of ARF6-regulated ERK activation, to facilitate TMV release^17^. Given that TMV release is also strongly enhanced by the activation of RhoA or the suppression of Rac1, we investigated if a similar pathway functioned during TMV release under these conditions. To this end, we examined MLC and ERK phosphorylation in cells either expressing RhoA(G14V) or pharmacologically inhibited for Rac1 activation by NSC23766 treatment. Increases in both MLC and ERK phosphorylation were observed under both experimental conditions (Fig. 7a). We also determined that TMV shedding was dependent on ERK activity, as co-treatment with the MEK1/2 inhibitor U0126 significantly diminished microvesicle shedding under both experimental conditions (Fig. 7b,c). Notably, however, the selective inhibition of MLCK by treatment with ML-7 did not prevent this upregulated TMV release (Fig. 7c). This suggests upon Rac1 inhibition and concurrent RhoA activation, there may be alternate mechanisms responsible for the increase in MLC phosphorylation for TMV shedding, which are independent of MLCK.

In light of the above findings, we investigated whether the regulation of myosin light chain phosphatase would lead to MLC activation to facilitate TMV shedding upon Rac1 inhibition. Myosin light chain phosphatase (MLCP) counters the activity of MLCK, to inactivate MLC^33^,^34^. MLCP itself can be inhibited by phosphorylation of threonines 696 and 853 of its targeting subunit, MYPT1; events known to be regulated by ROCK and ERK^35^,^36^. Indeed, upon pharmacological inhibition of Rac1, a significant increase in phosphorylation is seen at threonines 696 and 853 of MYPT1 (Fig. 7d). Inhibition of ERK consistently decreased phosphorylation of MYPT1 Thr853 and Thr696, whereas inhibition of ROCK consistently decreased phosphorylation of MYPT1 Thr853 but not at site Thr696. Inhibition of ERK or ROCK was sufficient to significantly attenuate shedding of TMVs. The phosphorylation of MLCP likely occurs in response to Rac1 inhibition, because relative to parental LOX or cells expressing activated ARF6, the level of phosphorylated MYPT1 Thr853 is drastically upregulated in cells treated with NSC23766 (Fig. 7e). Thus, Rac1 inhibition prompts the activation of Rho—ROCK—MLCP pathway for TMV release (Fig. 7f). In sum, MLCP inactivation is another means through which myosin contractility regulates the formation of tumor microvesicles.

## Discussion

In this study we present data that demonstrates how tumor cells can switch between the use of invadopodia and TMVs to successfully invade through extracellular matrices. We have found that signaling through either Rac1 or RhoA is responsible for promoting the formation of invadopodia or microvesicles, respectively. While tumor cells can form both invasive structures, antagonistic signaling through these pathways favors the formation of one structure at a given time. Increased Rac1 activity facilitates invadopodia formation in cells encountering a firm matrix, however Rac1 activation is not sufficient to promote invadopodia formation on softer, more deformable matrices. This is in concert with existing reports that matrix rigidity markedly influences the formation of invadopodia^27^,^37^, as does matrix cross-linking and fiber density^38^. Similarly, while more compliant matrix environments promote microvesicle formation through RhoA signaling, firm matrices do not support TMV shedding. Through these signaling pathways, tumor cells may switch between mesenchymal and amoeboid modes of movement as previously described^7^,^39^,^40^, permitting invasion through different microenvironments, such as dense connective tissue versus a soft adipose matrix, which may be encountered as a cell moves from a tumor toward a metastatic site.

That firm and compliant matrices favor the formation of invadopodia and microvesicles, respectively, is also manifested in the cellular Rac1 and RhoA activities in these respective environments. While Rac1-GTP pools are significantly higher in cells invading firmer matrices, Rho activation is elevated in amoeboid cells on compliant matrices. Furthermore, we also show that microvesicles contain abundant RhoA. Further work will be necessary to elucidate the mechanisms regulating the ability of cells to toggle between these two modes of invasion, which likely requires the engagement of specific Rac1 and RhoA regulatory molecules such as GTPase-activating proteins (GAPs) and guanine nucleotide exchange factors (GEFs) to facilitate enhanced activation of either Rac1 or RhoA. These data support published work indicating a role for RhoA in extracellular vesicle formation^41^, however at this time it is unclear if the same vesicle populations are being evaluated as in that study, as RhoA was not found to be present on shed vesicles in the previous study. Of further note, another study has shown that the release of exosomes promotes invadopodia-mediated cell invasion^42^. While we have not investigated the role of exosomes here, it underscores the dynamic nature of invadopodia and the importance of the different types of extracellular vesicles in tumor cell invasion.

The studies described here further attest to the importance of actomyosin-based contractility in TMV release. While earlier studies have documented a role for MLC activation via myosin light chain kinase, we show that inhibition of myosin light chain phosphatase may also be promoting MLC activation in these cells that display elevated TMV formation. Inhibitory phosphorylation of the MYPT1 subunit of MLCP is markedly enhanced upon downregulation of Rac1 activity. This Rho-driven regulation of microvesicle formation lies downstream of ARF6, as the inhibition of ROCK in cells expressing constitutively-active ARF6 is sufficient to significantly attenuate TMV shedding in deformable matrices. Further, the over-expression of active RhoA in cells in which TMV formation is blocked by the expression of dominant-negative ARF6 is sufficient to induce microvesicle release. While ROCK-mediated actomyosin-based contractility regulates TMV release, it occurs independently of MLCK, as blocking MLCK activity had little to no effect on shedding mediated by the constitutive activation of RhoA or pharmacological down regulation of Rac1 activation, although it efficiently blocks TMV shedding downstream of constitutively activated ARF6. We propose that the Rho-ROCK-MLCP pathway may be specifically upregulated in tumor cells as they encounter certain extracellular cues (such as compliant environments) that prompt the downregulation of Rac1 signaling along with a concomitant up-regulation of RhoA activation.

A recent study has brought to light the idea that the activation of Rac1 may promote invadopodia disassembly^43^. While this study highlights the importance of Rac1 cycling in invadopodia turnover which is required for efficient cell invasion, how these findings relate to the data described here remain to be determined, but will be important to better understand the interplay of Rho GTPase signals and cell invasion. Another study has shown that ARF1 activation can promote invadopodia-mediated invasion and microvesicle formation in invasive breast tumor cell lines^44^. Notably, constitutive ARF1 activation had no effect on these behaviors in melanoma cells (Supplementary Fig. S4,5), suggesting that ARF1 activation may not be involved in the pathways described here. There are likely to be many proteins which modulate the conversion between microvesicle- or invadopodia-mediated cell invasion including, for example, Cdc42 with its well-characterized role in invadopodia biogenesis^45^, or various other actin and myosin regulatory proteins involved in cell protrusion and motility, all of which provide interesting avenues for further research.

Analyses of melanoma tumors have shown cells appearing as both rounded and elongated^5^,^32^,^46^, and recent work in mammary tumors has highlighted invasive modes that cells may use during invasion^47^. The data described in this study provide insight into the plasticity of tumor cell invasion, wherein cells interchangeably adopt distinct morphologies in a process governed by the specific extracellular milieu encountered. Cells in firm environments maintain a mesenchymal morphology with a corresponding use of invadopodia to proteolyze matrix, whereas rounded cells utilize an amoeboid mode of motility and the release of microvesicles for distal discharge of proteases when a more compliant environment is encountered. These findings build upon work from others which highlight the importance of proteolysis in enhancing the invasion of both mesenchymal and amoeboid type cells^5^,^30^. As tumor cell dissemination is a highly variable process, encountering different cell types and cues from the microenvironment may alter these signaling events^48^. We propose that efficient tumor cell dissemination during metastasis will utilize each mode of invasion, depending on the cellular environment. Consequently, targeting both modes of invasion will maximize the efficacy of therapeutics designed at reducing tumor cell invasive capacity.

## Methods

### Cell culture and transfection

Parental LOX and LOX cells stably expressing ARF6(Q67L) or ARF6(T27N) and referred to as LOX^ARF6-GTP^ and LOX^ARF6-GDP^, respectively^15^, were grown in RPMI; SW480 were grown in Leibovitz’s L-15; and PC-3 were grown in Ham’s F-12K. All media was supplemented with 2 mM l-glutamine (Gibco), penicillin/streptomycin (Thermo Scientific), and 10% FBS (Hyclone), with the exception of LOX-ARF6 mutants which were grown in tetracycline-free FBS (Clontech), in the presence of G418 (Gibco) and hygromycin (Invitrogen). FBS used for microvesicle isolations was pre-cleared of microvesicles and exosomes as detailed in “Microvesicle Isolation” below. LOX cells were transfected using GeneExpresso (Excellgen) according to the manufacturer’s suggested protocol.

### Antibodies, plasmids and other reagents

pCGT-Rac1(T17N) and pCGT-Rac1(G12V) were gifts from Dr. Linda Van Aelst (CSHL), and pCGT-RhoA(G14V) and pCGT(T19N) were gifts from Dr. Alan Hall (Memorial Sloan Kettering Institute). pcDNA-HA-ARF1(T31N) (plasmid #10833) and pcDNA-HA-ARF1(Q71L) (plasmid #10832) were purchased from Addgene, deposited by Thomas Roberts. The following antibodies were utilized for these studies: Mouse anti-T7 (Novagen); rat anti-β_1_ integrin clone AIIB2 (Iowa DSHB); mouse anti-RhoA (Cytoskeleton); mouse anti-α-tubulin (Sigma); mouse anti-paxillin (BD Biosciences); mouse anti-HA (Covance); rabbit anti-cortactin, rabbit anti-cleaved caspase-3, rabbit anti-pMLC^18/19^, rabbit anti-MLC, mouse anti-pERK1/2^202/204^, rabbit anti-ERK1/2, rabbit anti-MYPT1/anti-MYPT1^Thr696^/anti-MYPT1^Thr853^ (all from Cell Signaling). The ARF6 antibody is a mouse monoclonal generated within the lab. Phalloidin conjugates were purchased from Molecular Probes. NSC23766 was used at 50 μM, ML-7 at 10 μM, and Y-27632 at 10 μM, and all were purchased from Calbiochem. U0126 was used at a 20 μM concentration and purchased from LC Laboratories.

### Cell invasion assays

FITC-conjugated gelatin coverslips was prepared as described previously^49^. Briefly, 18 mm round glass coverslips (Fisherbrand) were coated with gelatin by micropipette, and gelatin layers were ≤5 μm for a firm and thin coating and greater than 20 μm for a thick and complaint coating. These matrices were crosslinked using 0.5% glutaraldehyde. Unless indicated otherwise, cells were allowed to invade for approximately 16 hours prior to fixation and staining. All inhibitor treatments were initiated after the cells had been allowed to adhere for one hour after plating. For the quantification of invadopodia-mediated degradation, the number of cells localized over matrix degradation was visualized via microscopy and calculated as a percentage of the total number of cells. 100 cells were counted per condition, compiled from 3 independent experiments. For the quantification of TMV release, the number of free microvesicles was quantified by microscopy. Fields were chosen at random, moving systematically through the coverslip, isolated cells were identified, and the number of β_1_ integrin positive microvesicles clearly detached from the cell, within one cell’s diameter distance from the periphery, were counted by focusing through all planes of the matrix. 140 cells were counted per condition unless otherwise noted, compiled from 3 independent experiments. Error bars represent the standard error of the mean. Statistical analysis was accomplished utilizing Microsoft Excel and GraphPad Prism software. The two-tailed unpaired t-test (Figs 3F and 5E), Kruskal-Wallis H test (with Dunn’s test) (Figs 3B, 5B, 5H, 7C and S3), and Mann-Whitney U tests (Figs 1E and 6B) were used to assess statistical significance. Probability levels <0.05 were considered statistically significant. For experiments utilizing collagen, cells were grown on type I bovine collagen (PureCol from Advanced Biomatrix) using the manufacturer’s suggested protocol for 3-D gel preparation, in 35 mm glass-bottom dishes (MatTek), which had been coated with poly-L-lysine. Cells were fixed and stained as detailed below, and imaged using a Nikon A1R microscope equipped for confocal reflection microscopy. For the assessment of cells in varied concentrations of gelatin, porcine skin gelatin (Sigma) was dissolved in warm PBS and applied to coverslips using the same methods as for the FITC-gelatin slips.

### Immunofluorescence staining

Cells were fixed and stained as described previously^15^, and mounted using Prolong Gold Antifade mounting medium (Invitrogen). Imaging was performed using a BioRad MRC-1024 laser scanning confocal microscope or a Zeiss Observer Z1 widefield fluorescence microscope. Image processing was performed using Image J (NIH).

### Live imaging

Live videos were obtained using an Andor Revolution Spinning Disk confocal microscope equipped with a Tokai Hit environmental chamber. Cells were grown in 35 mm dishes with glass coverslip bottoms (MatTek) in phenol-free media (Hyclone), and images were obtained every 30 seconds over the course of several hours. Image processing was performed using Image J (NIH).

### Microvesicle isolation

Isolation of tumor cell-derived microvesicles and exosomes was performed as previously described^17^, with minor modification. Microvesicles were isolated by spinning at 10,000 × g for 1 hour, and were washed with PBS 3 times prior to lysis into RIPA buffer containing mammalian protease inhibitor cocktail (Sigma), prior to western analysis.

### Western blotting and GTPase activation assays

Where indicated, cells were grown on coverslips coated with a thin or thick layer of gelatin for 16 hours prior to lysis. Otherwise, cells were cultured on tissue culture plastic. For western blotting, cells were lysed in RIPA buffer containing mammalian protease inhibitor cocktail (Sigma), and protein was quantified when required (BCA assay from Thermo Scientific). Phosphatase inhibitor cocktail (Alfa Aesar) was added to the lysis buffer of samples that were to be evaluated for phosphorylated proteins. The PAK-PBD effector pulldown assays were carried out as previously described^50^. The Rhotekin-RBD effector assays were carried out using a prepared kit from Cytoskeleton, following the manufacturer’s suggested protocol.

### Nanoparticle tracking analysis

For quantification of microvesicles released from cells in varied matrix conditions, conditioned media was harvested and processed as in Microvesicle Isolation above. Pelleted microvesicles were resuspended in PBS and analyzed via nanoparticle tracking using a NanoSight LM10 (NanoSight Ltd., Salisbury, UK).

## Additional Information

**How to cite this article**: Sedgwick, A. E. *et al.* Extracellular microvesicles and invadopodia mediate non-overlapping modes of tumor cell invasion. *Sci. Rep.* **5**, 14748; doi: 10.1038/srep14748 (2015).

## Supplementary Material

Supplementary Information

Supplemetal Movie 1

Supplemetal Movie 2
