# Supplementary material for: Extracellular microvesicles and invadopodia mediate non-overlapping modes of tumor cell invasion
Source: Sci Rep. 2015 Oct 13;5:14748. doi: 10.1038/srep14748 (PMC4602187; doi:10.1038/srep14748)
Supplement: Supplementary Information [file srep14748-s1.pdf]

Supplemental Figures

Extracellular microvesicles and invadopodia mediate non-overlapping modes of tumor cell invasion

Alanna E. Sedgwick, James W. Clancy, M. Olivia Balmert, Crislyn D'Souza-Schorey*

Department of Biological Sciences, University of Notre Dame, Notre Dame, IN 46556-0369, USA

*To whom correspondence should be addressed. Email: cdsouzas@nd.edu; Tel: 574-631-3735

Figure legends: Supplemental figures

S1. Cells release TMVs in a fibrillar collagen environment. Cells were grown on a type I collagen matrix prior to fixation and staining to visualize β_1 integrin (green). Collagen was imaged using confocal reflection microscopy, and is pseudocolored red.

S2. Visualization of isolated TMVs. Microvesicles were isolated from cells as outlined in the Methods. They were overlaid onto poly-L-lysine-coated coverslips prior to fixation and staining to visualize β_1 integrin. Scale bar 20 µm.

S3. Matrix concentration modulates microvesicle release. Microvesicles released from cells grown on matrices of 1-5% gelatin were quantified by microscopy, as described in the Methods. 50 cells were counted per experimental condition. The fold change in average number of TMVs released relative to that in 1% gelatin, is plotted. Error bars represent the standard error of the mean.

S4. ARF1 activation status does not impact invadopodia-mediated melanoma invasion. Cells transfected with ARF1(T31N) or ARF1(Q71L) were grown on a thin layer of FITC-gelatin prior to fixation and staining to visualize the HA tag on the ARF1 mutant (pseudocolored cyan) and filamentous actin (red). Optical sections along the ventral cell surface are shown. Arrows point to transfected cells.

S5. ARF1 activation status does not impact TMV shedding from melanoma cells. Cells transfected with ARF1(T31N) or ARF1(Q71L) were grown on a thick layer of unlabeled gelatin prior to fixation and staining to visualize the HA tag on the ARF1 mutant (pseudocolored cyan), filamentous actin (red), and β_1 integrin (green). Arrows point to transfected cells.

Movie Legends

Movie 1. Melanoma cell invasion on thin/firm gelatin matrix. Cells were transiently transfected to express cytoplasmic mCherry fluorescent protein and grown on a thin layer of FITC-gelatin. Cells were imaged live using confocal fluorescence microscopy as outlined in the Methods. Images were taken every 30 seconds and movies presented at 45 frames per second. Cells can be seen to proteolyze punctate foci as they move across the matrix in a mesenchymal morphology.

Movie 2. Melanoma cell invasion on thick/deformable gelatin matrix. Cells were transiently transfected to express cytoplasmic mCherry fluorescent protein and grown on a thick layer of FITC-gelatin. Cells were imaged live using confocal fluorescence microscopy as outlined in the Methods. Images were taken every 30 seconds and movies presented at 7 frames per second. Cells can be seen actively blebbing and clearing tracks as they move through the matrix in a rounded morphology.

Supplemental Figures

S1.

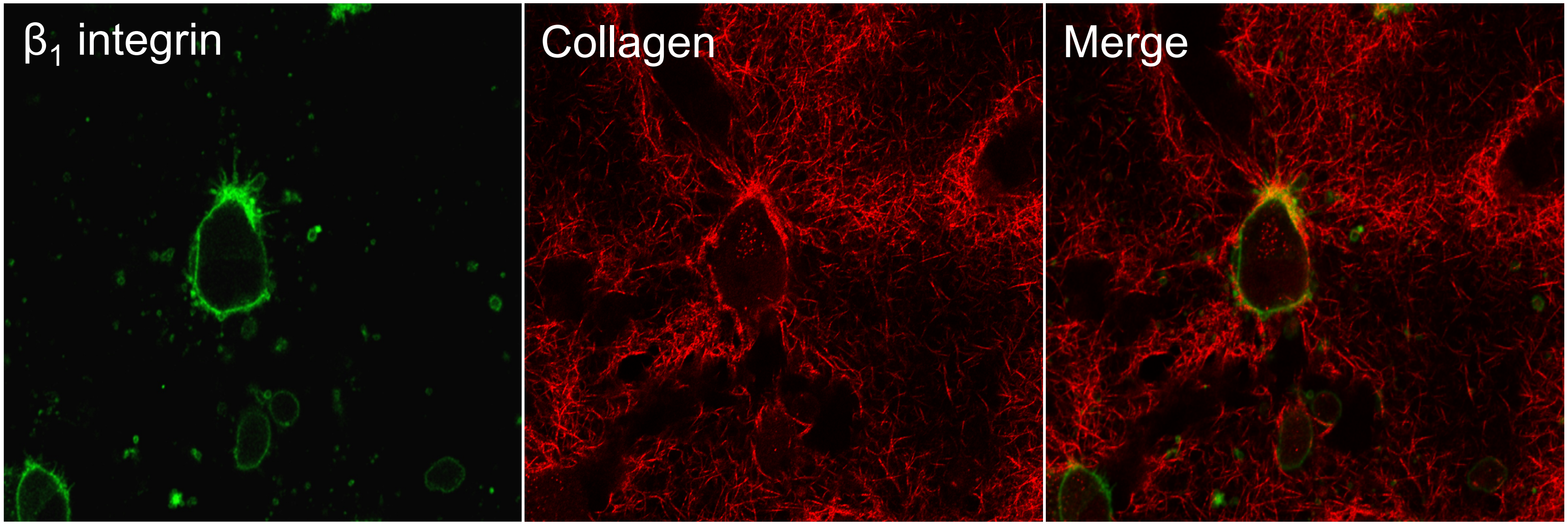

S2.

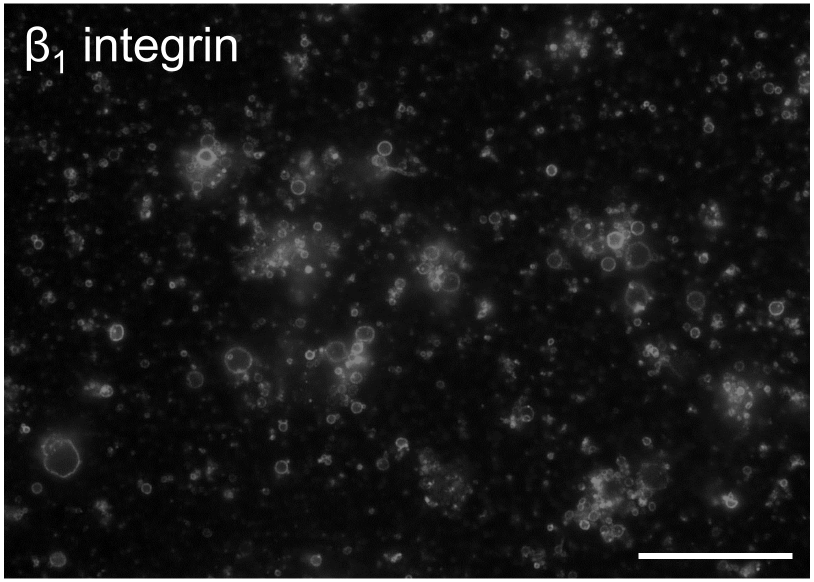

S3.

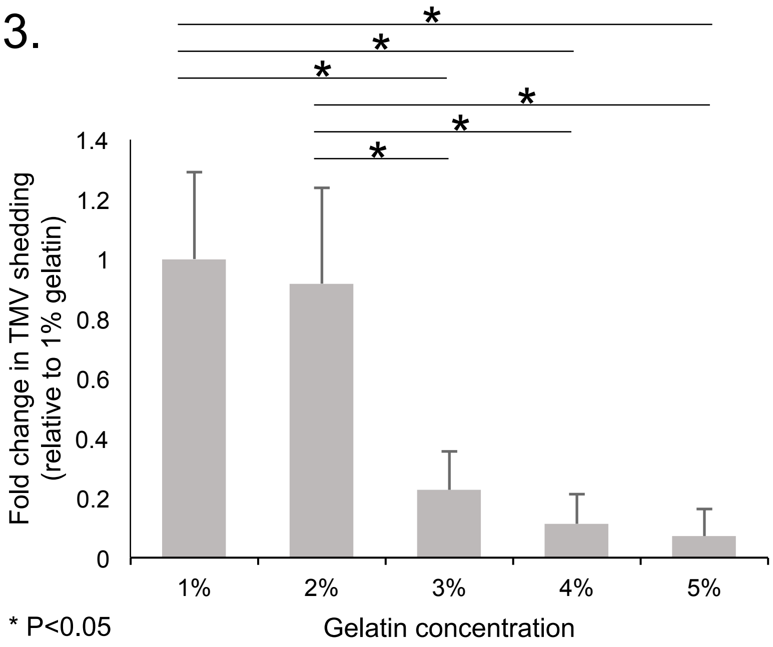

Sedgwick et al.

Supplemental Figures

Gelatin Merge

S4.

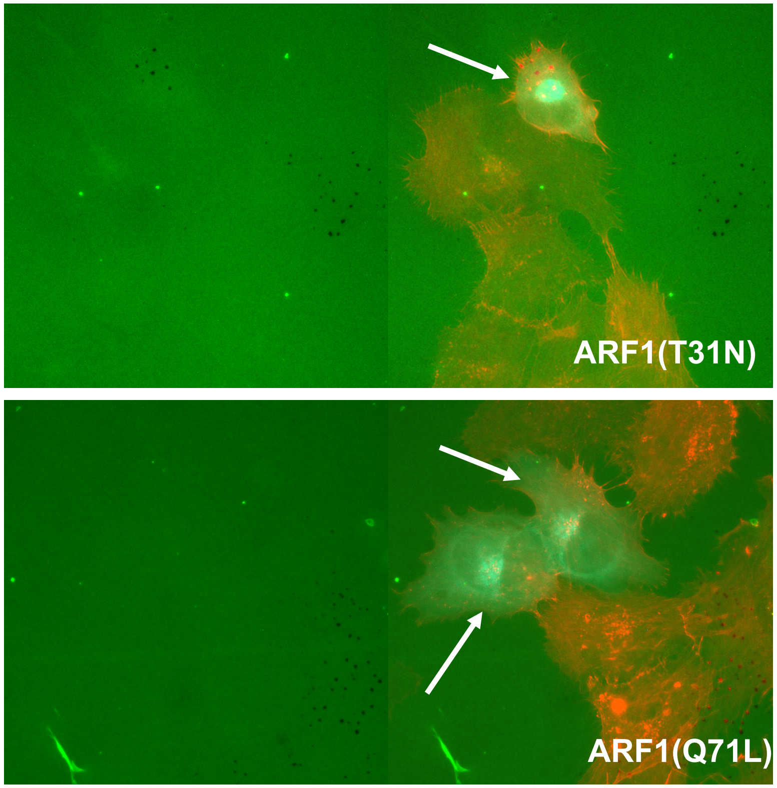

Actin HA-ARF1 Gelatin

S5.

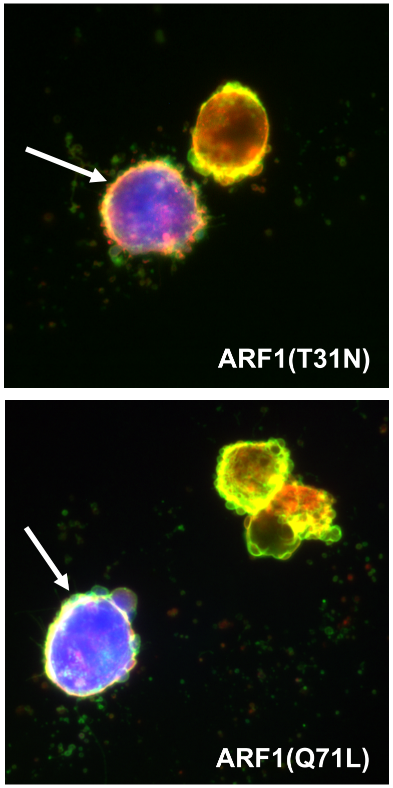

Actin HA-ARF1 Gelatin

Sedgwick et al.
